# Posterior Capsule Opacification after Cataract Surgery in Children Over Five Years of Age with Square-edge Hydrophobic *versus* Hydrophilic Acrylic Intraocular Lenses: A Prospective Randomized Study

**DOI:** 10.6061/clinics/2020/e1604

**Published:** 2020-04-30

**Authors:** Camila Ribeiro Koch, Marcony R Santhiago, Priscilla A Jorge, Paulo Sena, Newton Kara-Júnior

**Affiliations:** I Departamento de Oftalmologia, Faculdade de Medicina FMUSP, Universidade de Sao Paulo, Sao Paulo, SP, BR; II Hospital Humberto Castro Lima, Salvador, BA, BR; III University of Southern California Roski Eye Institute, Los Angeles, CA, USA; IV Departamento de Oftalmologia, Universidade Federal do Rio de Janeiro, Rio de Janeiro, RJ, BR

**Keywords:** Posterior Capsule Opacification, Pediatric Cataract, Hydrophilic IOL, Hydrophobic IOL, EPCO

## Abstract

**OBJECTIVE:**

To compare the effects of hydrophobic and hydrophilic materials in square-edged acrylic intraocular lenses (IOLs) on the development of posterior capsule opacification (PCO) after pediatric cataract surgery.

**METHODS:**

Patients were randomly assigned to group 1 (hydrophobic acrylic square-edged IOLs; 13 eyes) or group 2 (hydrophilic acrylic square-edged IOLs; 13 eyes). The study evaluated PCO rates using Evaluation of Posterior Capsule Opacification (EPCO) 2000 software at one, three, six and 12 months postoperatively. Postoperative measurements also included corrected distance visual acuity (CDVA), neodymium:yttrium‐aluminum‐garnet (Nd:YAG) capsulotomy and postoperative complications other than PCO.

**RESULTS:**

Both groups had significant increases in PCO rates after one year. Comparison of the groups showed no significant differences in the EPCO scores at three (group 1, 0.007±0.016 *vs* group 2, 0.008±0.014; *p*=0.830), six (group 1, 0.062±0.103 *vs* group 2, 0.021±0.023; *p*=0.184), or twelve months postoperatively (group 1, 0.200±0.193 *vs* group 2, 0.192±0.138; *p*=0.902). We also found no significant group differences regarding the change (delta, Δ) in EPCO scores between three and six months (group 1, 0.055±0.09 *vs* group 2, 0.013±0.02; *p*=0.113) or between six and twelve months postoperatively (group 1, 0.139±0.14 *vs* group 2, 0.171±0.14; *p*=0.567). Twenty-three percent of patients required Nd:YAG capsulotomy at the twelve-month visit.

**CONCLUSIONS:**

No differences in PCO rates were found between hydrophobic and hydrophilic acrylic square-edged IOLs in children between five and twelve years of age at one year of follow-up.

## INTRODUCTION

The ideal intraocular lens (IOL) to implant in children should have good uveal and capsular biocompatibility 1-2 because the growth of the residual lens epithelial cells remaining after cataract surgery is faster in children than in adults, with an increased inflammatory response in this age group 3-5. Hydrophilic acrylic square-edged IOLs may benefit children because these lenses have good uveal biocompatibility, and the presence of the square edge might compensate for the relatively low capsular biocompatibility 1-6.

The introduction of IOLs with a sharp edge design has improved surgical outcomes by inhibiting lens epithelial cell migration and proliferation through a mechanical barrier effect that reduces posterior capsule opacification (PCO) rates in adults 7-13. Most studies involving adults have found higher PCO rates in those with hydrophilic acrylic IOLs than in those with hydrophobic acrylic IOLs; however, many of these studies have included hydrophilic acrylic IOLs with round edges 1-14. Moreover, it may not be appropriate to generalize these results to children because the tissue reactivity in children is different than that in adults. In addition to the biocompatibility of the materials, postoperative complications and costs should also be considered when choosing IOLs 15. The settings of some countries may influence the choice of IOL types, as hydrophobic IOLs typically have a higher cost than hydrophilic IOLs.

Although IOL implantation has become increasingly common in pediatric cataract patients, literature evaluating the materials from which IOLs are made is scarce. Few publications have compared polymethylmethacrylate (PMMA) *vs* acrylic hydrophobic IOLs 16-18 and PMMA *vs* hydrophilic IOLs; furthermore, most of the evaluated IOLs do not have square edges 19. One recent retrospective study comparing hydrophobic *vs* hydrophilic IOLs in children 20 showed comparable visual and surgical outcomes and equal rates of PCO. Therefore, this prospective study aimed to compare the effects of hydrophobic *vs* hydrophilic acrylic square-edged IOLs for primary in-the-bag implantation in pediatric cataract patients on the development of PCO at 1 year using the Evaluation of Posterior Capsule Opacification (EPCO) 2000 system for the first time. Visual outcomes and other complications were also documented.

## PATIENTS AND METHODS

This prospective randomized study included children between five and twelve years of age with unilateral or bilateral developmental cataracts who underwent cataract surgery with IOL implantation at the Humberto Castro Lima Hospital in Salvador, Brazil. Between January 2016 and December 2016. Informed consent was obtained from the parents/legal guardians of all participants prior to enrollment. The study followed the tenets of the Declaration of Helsinki and was approved by the Medical Institutional Review Board with the approval of the University of São Paulo, São Paulo, Brazil. It is registered on the National Institutes of Health Clinical Trials website.^A^

The exclusion criteria were coexisting ocular disease, systemic diseases, secondary cataracts (trauma, uveitis) and previous ocular surgery. Patients with poor cooperation due to neurological delays or difficulty attending follow-up examinations due to distance (residence more than 250 km from the hospital) were also excluded. Children under five years of age were excluded because intraoperative capsulotomy is routinely performed in this age group.

Patients were randomly assigned to either group 1 (hydrophobic acrylic square-edged IOLs) or group 2 (hydrophilic acrylic square-edged IOLs) according to assigned numbers from a computer-generated randomization program. For bilateral cataracts, the first operated eye was included in the randomized group, and the second eye was included in the other group. Parents/legal guardians and patients were blinded to the assigned interventions. The sample size was estimated based on the results of a previous clinical trial (mean±standard deviation (SD) of the EPCO score: hydrophobic group: 0.0±0.13 *vs*. hydrophilic group: 1.3±1.35) 21, considering a 95% confidence level and 80% power. To detect a difference of -1.3 in the EPCO score, the required sample size was nine patients per group.

### Preoperative Assessment

One of two experienced examiners, both of whom were blinded to the assigned interventions, performed a complete examination, which included an assessment of visual acuity (VA), Goldmann applanation tonometry, manifest refraction and fundoscopy for each child. The types of lens opacities were assessed with a slit lamp after the induction of mydriasis 22.

### IOLs

The IOL models compared in this study differed in acrylic material compositions and configurations. Patients in group 1 were treated with an AcrySof SA60AT model (Alcon Laboratories, Fort Worth, TX, USA), which is a hydrophobic (acrylate-methacrylate copolymer) IOL, monofocal, 6.0 mm optic diameter, that is 13.0 mm in overall length, with 0° haptic angulation, a spherical shape and a continuous square edge, except at the haptics. Patients in group 2 were treated with an Akreos Adapt Advanced Optics (AO) model (Bausch & Lomb, Rochester, NY, USA), which is a hydrophilic (polyhydroxyl-ethyl methacrylate/methyl methacrylate copolymer) IOL, monofocal, 6.0 mm optic diameter, 10.5 to 11.0 mm in overall diameter, with an aspheric shape and a 360° posterior barrier edge, except at the 4 optic-haptic junctions. All IOLs were biconvex and commercially available sharp-edged designs and foldable single-piece units with an ultraviolet (UV) light filter 21,23-26.

### IOL Power Calculation

The Sanders-Retzlaff-Kraff/theoretical (SRK/T) formula was used for the IOL power calculation. Immediate postoperative undercorrection was planned according to the patient's age: +1 diopter (D) in children between 5-6 years of age and emmetropia in patients older than seven years of age.

### Surgical Procedure

All children underwent cataract surgery under general anesthesia. The pupil was dilated with 1% cyclopentolate and 2.5% phenylephrine. One experienced surgeon (CK) performed all surgeries in both groups using a standardized surgical procedure in both groups. In brief, a temporal 2.75-mm self-sealing clear corneal incision was made, and trypan blue was injected. A balanced salt solution (BSS, Alcon, Fort Worth, TX, USA) containing an epinephrine (1 ml/L) wash and an ophthalmic viscosurgical device (OVD) (Ophthal-Fill HPMC 2%) were injected into the anterior chamber. A manual anterior continuous curvilinear capsulorhexis of 5.0 mm in diameter was created with Utrata forceps. Hydrodissection was performed, followed by irrigation/aspiration (I/A) of the lens and cortex and polishing of the anterior capsule. After insertion of the OVD, the IOL was inserted in-the-bag through the main incision with an injector, the OVD was aspirated using I/A, and acetylcholine was injected into the anterior chamber. At the end of the procedure, an inferior subconjunctival steroid injection (dexamethasone 0.4 mg) was administered, and antibiotic drops were administered in the operated eye. No sutures were used. There was a 15-day interval between surgeries in bilateral cases.

The standardized postoperative regimen was as follows: 0.5% moxifloxacin eye drops (Vigamox, Novartis International AG, Switzerland) were administered every six hours for one week; 1% prednisolone acetate eye drops were administered every four hours for one week and then tapered gradually over four additional weeks; and 1% tropicamide eye drops were administered twice daily for two weeks.

### PCO

Patient evaluations were performed postoperatively at one, seven and 30 days; once monthly for up to six months; and every three months thereafter. Digital retroillumination images of the posterior capsule of each operated eye were obtained at one, three, six and 12 months after surgery through maximally dilated pupils (tropicamide and phenylephrine eye drops were administered once every 10 minutes for 3 cycles) using a high-resolution digital camera (Canon EOS 6D with a Canon SLR/D-SLR adapter) mounted on a slit lamp (Huvitz) under fixed illumination. In those patients who required capsulotomy, digital retroillumination images of the posterior capsule were taken at the same visit but before the Nd:YAG was performed. The PCO images were imported into EPCO 2000 image analysis software. As children have an increased risk for capsular phimosis and opacity of the anterior capsule, the EPCO was conducted within the capsulorhexis area rather than according to the optic size of the IOL. The capsulorhexis border was marked, after which the PCO areas were drawn manually. To avoid interfering with the results, only IOL optics (without the haptics) were presented to the examiner to mark the capsulorhexis in the EPCO program. Dense areas were identified and actively marked on the computer screen by the observer. The individual PCO score for each eye was calculated by EPCO, multiplying the density of the opacification (graded 0 to 4) by the fractional PCO area behind the IOL optic. The EPCO program calculated the density surface mathematically by performing pixel counts. The EPCO score (scale, 0 to 4) and EPCO area (mm^2^) were interpreted by the software. The density of the opacification was also clinically evaluated with a slit lamp and subjectively graded as 0 (none), 1 (mild), 2 (moderate) or 3 (severe) at the final visit.

Our primary outcome measures were the mean EPCO scores at one, three, six and 12 months. We also investigated the delta (Δ) values of the EPCO scores between three and six months and between six and 12 months.

### Other Outcome Measures

Postoperative complications, refraction changes, corrected distance visual acuity (CDVA) at the one-year follow-up and neodymium:yttrium‐aluminum‐garnet (Nd:YAG) capsulotomy rates were also recorded. Nd:YAG laser capsulotomy was performed in patients with decreased VA (one or more lines) according to the clinical evaluation of PCO. If the patient underwent capsulotomy, the EPCO score was not applied. The EPCO score was applied to patients before Nd:YAG laser capsulotomy was necessary. Macular optical coherence tomography (Zeiss OCT) was performed in all patients within the first month after surgery. Intraocular pressure was measured at all visits.

### Statistical Analysis

All quantitative data were found to be normally distributed according to the Kolmogorov-Smirnov test and descriptive statistics. Quantitative variables are expressed as means and SDs. Qualitative variables are expressed as absolute and relative frequencies. Independent *t*-tests were used to compare means between the groups. For intragroup comparisons of the means at four timepoints (one month, three months, six months and 12 months), repeated measures ANOVA was used. To compare the mean EPCO scores between three and 12 months and to evaluate the intragroup EPCO score variation, paired *t*-tests were used. Statistical Package for the Social Sciences (version 19.0, SPSS, Inc., Chicago, USA) for Windows software was used for the statistical analysis. A *p*-value<0.05 was considered statistically significant.

## RESULTS

Twenty-eight eyes (19 patients) were included in the study. Twenty-six eyes (13 eyes in each group) were included in the final analysis. Two selected children (both with unilateral cataracts) were excluded from the analysis; one moved to another city, and the other required an intraoperative posterior capsulotomy because of a dense cataract with involvement of the posterior capsule. Eight patients (8 eyes) with unilateral cataracts and nine patients (18 eyes) with bilateral cataracts were analyzed. No other intraoperative complications were observed. All patients analyzed underwent IOL in-the-bag implantation.

### Preoperative Demographics

No significant differences were observed in the mean age at surgery or the gender distribution between the groups (*p*=0.497 and *p*=0.619). The mean age at surgery was 7.04±2.47 SD (range, 5 to 12) years. Of the children analyzed, seven children (13 eyes) were five years old when they underwent surgery, three children (3 eyes) were seven years old, three children (4 eyes) were eight years old, one child (1 eye) was nine years old, one child (2 eyes) was ten years old and two children (3 eyes) were twelve years old. The most frequent cataract morphology was nuclear (16, 61.5%), followed by lamellar (5, 19.2%). The mean axial length was 23.30±1.26 mm. Table 1 shows the demographic data of the patients in both groups.

### Main Outcomes

**Figure 1:**
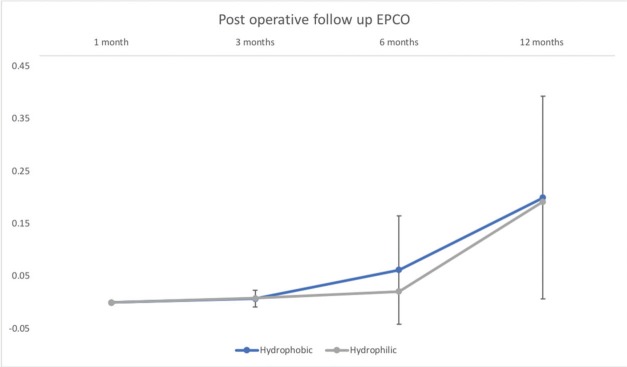
Evaluation of the Posterior Capsule Opacification (EPCO) score by group during the follow-up period. At one month: group 1 (0.007±0.016) *vs* group 2 (0.008±0.014) (*p*=0.830); at six months: group 1 (0.062±0.103) *vs* group 2 (0.021±0.023) (*p*=0.184); and at twelve months: group 1 (0.200±0.193) *vs* group 2 (0.192±0.138) (*p*=0.902).

A significant increase in the PCO rate was observed in both groups (*p*<0.05) at the one-year follow-up. Table 2 shows the comparisons between the EPCO scores at three, six and twelve months within and between the two groups. The EPCO score was available for all eyes included in the study. The mean EPCO score at one month was 0.0 in both groups. No statistically significant difference was found when the PCO rates were compared between the two groups (*p*=0.881) at the one-year follow-up or at the final follow-up (*p*=0.902). The EPCO score evaluation by group during the follow-up period is shown in Figure 1 (at three months, group 1 (0.007±0.016) *vs* group 2 (0.008±0.014) (*p*=0.830); at six months, group 1 (0.062±0.103) *vs* group 2 (0.021±0.023) (*p*=0.184); at twelve months, group 1 (0.200±0.193) *vs* group 2 (0.192±0.138) (*p*=0.902)).

The EPCO score change (Δ) between three and six months was 0.055±0.09 in group 1 and 0.013±0.02 in group 2 (*p*=0.113). The Δ between six months and twelve months was 0.139±0.14 in group 1 and 0.171±0.14 in group 2 (*p*=0.567).

**Figure 2:**
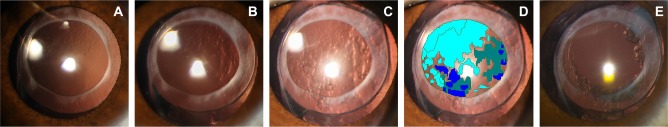
Evolution of pearl-type PCO in one eye that required treatment (A: three; B: six; and C and D: twelve months postoperatively. The opacification areas (D) were color coded according to their densities in the EPCO software (0: no color; I: light blue; II: intermediate blue; III: dark blue, IV: there were none in this case) and (E) after Nd:YAG capsulotomy.

No patient required capsulotomy prior to the 12-month postoperative visit. Six eyes (23.07%) required Nd:YAG capsulotomy at the twelve-month visit; four were in group 1 (*p*=0.097). However, no significant differences were observed between the two groups. No complications following capsulotomy were observed. Figure 2 illustrates the evolution and development of PCO in one eye that required treatment. Five eyes with PCO had pearl-type PCO, and one eye had fibrotic-type PCO. The clinical evaluations revealed mild PCO in nine eyes, moderate PCO in four eyes, severe PCO in one eye and no PCO in the remaining eyes at the last visit. Among patients who needed Nd:YAG capsulotomy, two eyes had other complications, one eye had synechiae, and one eye had pigment deposits. The mean VA in the six patients who needed capsulotomy after the procedure was 0.33 (0 to 0.6±0.26).

### VA and Refraction Outcomes

All the patients benefited from the surgery. The mean CDVA before surgery was 0.84±0.32 LogMAR. A significant improvement in VA was observed after surgery (*p*=0.000). One month after surgery, the mean CDVA was 0.27±0.18 LogMAR, and the mean spherical equivalent (SE) was 0.23±1.01 D. No statistically significant difference in CDVA was observed at the one-year follow-up between the two groups (*p*=0.675). The mean IOL power was 20.11±2.58 D.

### Other Complications

Other postoperative complications included posterior synechiae (two eyes in group 2), pigment deposits on the IOL (one eye in group 1 and two eyes in group 2) and IOL decentration (one eye in group 2) (*p*=0.160). Most of these complications occurred in children younger than six years of age at the time of surgery. The patient with IOL decentration required an additional surgery. No glaucoma was observed, and no cystoid macular edema was observed on macular optical coherence tomography. Additionally, in this study, no IOL glistening was noted, and there were no intraoperative challenges or complications.

## DISCUSSION

Regardless of efforts to delay the onset of PCO, it is a common complication following cataract surgery 19-27. PCO has a multifactorial pathogenesis, and patient age and IOL design play a crucial role in its development 28. This study compared the effects of two different commercially available acrylic square-edged IOLs on the PCO rates in children older than five years of age for the first time. Our study suggests that the IOL material did not influence the PCO rates in patients at the ages studied. These results differ from those of cataract surgery in adults, as PCO formation in children is much more aggressive than in adults; thus, the IOL design/material is likely to be less important than the surgical technique is in the prevention of PCO formation. In addition, this study observed that PCO tends to occur earlier in children, even in those over five years old, than in adults, as more than 20% of the evaluated eyes required Nd:YAG capsulotomy at the one-year postoperative visit.

In a retrospective study, Kleimann et al. 29 showed that 14% of children aged five months to 14 years old developed PCO at 47±21 months when a hydrophilic acrylic material was used; most of the implanted IOL models had a 360° square-edged design. Kleimann et al. suggested that a prospective study to compare PCO rates between hydrophilic and hydrophobic acrylic material in pediatric patients was needed because the existing literature on the topic is scarce.

Previous reports showed that square-edged IOLs prevented PCO 7-30. In adults, Kahraman et al. 24 reported a PCO rate of 0.23±0.36 as measured by EPCO software in adults at the three-year follow-up after implantation of the same hydrophobic acrylic IOL used in this study. In our study, a PCO rate of 0.20±0.19 was measured by EPCO software at the one-year follow-up. In addition, 23% of patients underwent Nd:YAG capsulotomy in the first year of follow-up; however, no significant differences were observed between the two groups. This result suggests that children develop PCO faster than adults, even when square-edged IOLs are used, which might be explained by the particular characteristics and behavior of children's eyes. Considering EPCO scores, Vasavada et al. 21 compared the same IOLs that we used in adults, and after one year, no statistically significant difference in the EPCO scores was found. During three years of follow-up, even though a higher PCO rate was observed in the hydrophilic group than in the hydrophobic group, only 16% of patients underwent Nd:YAG capsulotomy.

Regarding pediatric cataracts, only a few studies have compared PCO rates in patients treated with different IOL materials; moreover, only clinical PCO has been described. In a prospective study by Bhusal et al., children ranging in age from one month to eight years who underwent cataract surgery and primary posterior capsulotomy were examined. They found a higher number of PCO cases after treatment with hydrophobic acrylic IOLs than with silicone IOLs (both with square edges) after one year of follow-up; however, the difference was not significant 25. Panahi-Bazaz et al. 19 prospectively compared PMMA IOLs to hydrophilic acrylic IOLs with no sharp edges in children younger than six years old who underwent cataract surgery with capsulotomy and anterior vitrectomy. They reported PCO, which was not dependent on the IOL material used, in two children during more than one year of follow-up. Pigment deposits on the IOL were the only complication with a significant difference between the groups, exhibiting a lower rate in the acrylic group than in the PMMA group. Wilson 28, Ram 18 and Aasuri 31 compared PMMA and hydrophobic acrylic IOLs in a retrospective study and found lower PCO and uveal inflammation rates associated with acrylic IOLs than with PMMA IOLs. In our study, more cases of pigment deposits on the IOL were found in the hydrophilic group than in the hydrophobic group, even though the difference was not significant. Additionally, although statistical significance was not reached, more cases of synechiae and IOL decentration were also observed in this group. One possible reason for decentration is that hydrophilic acrylic IOLs are less rigid than hydrophobic acrylic IOLs.

In children older than five years, it is possible to leave the posterior capsule intact intraoperatively, and Nd:YAG laser capsulotomy can be performed if required because these patients have a lower amblyopia risk and lower PCO rates than those in younger children. The ideal age to perform an intraoperative capsulotomy has not yet been determined 5-32. In this study, 66.6% of Nd:YAG capsulotomies were performed in children younger than six years old, including those treated with both types of IOL materials, suggesting that younger children have higher rates of PCO than older children. Nd:YAG capsulotomies were successfully performed in these children. It may be interesting to perform posterior capsulotomy during surgery, at least in children undergoing cataract surgery at under six years of age. However, additional studies involving different IOL types and surgical techniques are needed to determine the optimal age group for this procedure.

As this was a pioneering study, the implanted IOLs and the age range of the included children were carefully selected. We chose IOLs for which the microstructure of the edge has already been studied microscopically. In addition, we included only children older than five years of age, as younger patients necessarily require intraoperative capsulotomy, which would prevent the documentation of PCO via the software 26. According to Werner et al., the square edges of hydrophilic IOLs have variable microedge structures, and this factor cannot be controlled for in prospective studies. A weakness of this study is the small number of eyes included; however, the sample size calculation procedure was followed. As such, the present conclusions should be interpreted with great caution.

The current study is the first to quantify PCO rates in children using software and prospectively compare two acrylic square-edged IOLs made of different materials. No significant difference in the PCO rate was observed between children treated with either type of IOL, but a large number of children required Nd:YAG capsulotomies, leading to the conclusion that intraoperative posterior capsulorhexis is a good option at least in children up to six years old. We will continue to follow these children for up to five years after their operations. In addition, an increased number of other complications was observed in the hydrophilic material group. Future clinical trials with an extended follow-up period, a larger number of children and different IOL designs are necessary.

## AUTHOR CONTRIBUTIONS

Koch CR substantially contributed to the conception and design of the study, data acquisition, analysis and interpretation, manuscript drafting and critical revision for important intellectual content and approval of the final version of the manuscript. Santhiago MR substantially contributed to the conception and design of the study, manuscript drafting and final approval. Jorge AP substantially contributed to the acquisition of the data, critical revision of the manuscript for important intellectual content and approval of the final version of the manuscript. Sena P substantially contributed to the conception and design of the study, data analysis and interpretation, drafting and critical revision of the manuscript for important intellectual content and approval of the final version of the manuscript. Kara-Júnior N substantially contributed to the conception and design of the study, drafting and critical revision of the manuscript and approval of the final version of the manuscript. All authors agree to be accountable for all aspects of the work and ensured that questions related to the accuracy or integrity of any part of the work are appropriately investigated and resolved.

## Figures and Tables

**Table 1:** Comparisons of the baseline demographic data between the groups.

	Mean±SD (Range)		
Parameter	Group 1	Group 2	*p*-value
Eyes (n)	13	13	_
Male n (%)	11 (84.6)	10 (76.9)	0.619**
Age at cataract surgery (y)	6.69±2.28 (5, 12)	7.38±2.69 (5, 12)	0.487*
IOL power (D)	20.50±2.48 (16.00, 24.50)	19.73±2.73 (14.50, 25.00)	0.460*
Axial length (mm)	23.07±1.27 (21.64, 25.16)	23.52±1.26 (21.79, 25.79)	0.374*
CDVA before surgery (LogMAR)	0.87±0.33 (0.5, 1.3)	0.83±0.33 (0.3, 1.3)	0.766*
CDVA at final follow-up (LogMAR)	0.29±0.18 (0.0, 0.6)	0.26±0.18 (0.0, 0.6)	0.675*
SE at final follow-up (D)	0.38±1.11 (-1.00, 2.75)	0.07±0.92 (-1.50, 1.50)	0.452*

SD=standard deviation; y=years; D=diopters; SE=spherical equivalent; CDVA=corrected distance visual acuity; mm=millimeters; IOL=intraocular lens; LogMAR=logarithm of the minimum angle of resolution;

** Chi-square;

* Independent *t*-test.

**Table 2:** EPCO score in each group during the follow-up period.

	Mean±SD		
Timepoint	Group 1 (13 eyes)	Group 2 (13 eyes)	*p*-value
3 months	0.007±0.016	0.008±0.014	0.830*
6 months	0.062±0.103	0.021±0.023	0.184*
12 months	0.200±0.193	0.192±0.138	0.902*
*p*-value	0.002α	0.000α	

SD=standard deviation;

* Independent *t*-test;

α =ANOVA test.

p* intergroup.

*p*^α^ intragroup.

## Footnotes

A U.S. National Institutes of Health Clinical Trials. Quantification of Posterior Capsule Opacification in Pediatric Cataract. Available from: https://clinicaltrials.gov/ct2/show/NCT02968290
